# Post-graduation migration intentions of students of Lebanese medical schools: a survey study

**DOI:** 10.1186/1471-2458-8-191

**Published:** 2008-06-02

**Authors:** Elie A Akl, Nancy Maroun, Stella Major, Claude Afif, Abir Abdo, Jacques Choucair, Mazen Sakr, Carl K Li, Brydon JB Grant, Holger J Schünemann

**Affiliations:** 1 Department of Medicine, University at buffalo, NY, USA; 2 Department of Social and Preventive Medicine, University at buffalo, NY, USA; 3 Department of Sociology, University at buffalo, NY, USA; 4 Division of Epidemiology, Public Health and Primary Care, Imperial College School of Medicine in London, UK; 5 Department of Medicine, University of Balamand, Beirut, Lebanon; 6 Department of Medicine, Lebanese University, Beirut, Lebanon; 7 Department of Medicine, Université Saint Joseph, Beirut, Lebanon; 8 Department of Medicine, Beirut Arab University, Beirut, Lebanon; 9 Dept. of Epidemiology & INFORMA, Italian National Cancer Institute Regina Elena, Rome, Italy

## Abstract

**Background:**

The international migration of physicians is a global public health problem. Lebanon is a source country with the highest emigration factor in the Middle East and North Africa and the 7th highest in the World. Given that residency training abroad is a critical step in the migration of physicians, the objective of this study was to survey students of Lebanese medical schools about their intentions to train abroad and their post training plans.

**Methods:**

Our target population consisted of all students of Lebanese medical schools in the pre-final and final years of medical school. We developed the survey questionnaire based on the results of a qualitative study assessing the intentions and motives for students of Lebanese medical schools to train abroad. The questionnaire inquired about student's demographic and educational characteristics, intention to train abroad, the chosen country of abroad training, and post-training intention of returning to Lebanon.

**Results:**

Of 576 eligible students, 425 participated (73.8% response rate). 406 (95.5%) respondents intended to travel abroad either for specialty training (330 (77.6%)) or subspecialty training (76 (17.9%)). Intention to train abroad was associated with being single compared with being married. The top 4 destination countries were the US (301(74.1%)), France (49 (12.1%)), the United Kingdom (31 (7.6%)) and Canada (17 (4.2%)). One hundred and two (25.1%) respondents intended to return to Lebanon directly after finishing training abroad; 259 (63.8%) intended to return to Lebanon after working abroad temporarily for a varying number or years; 43 (10.6%) intended to never return to Lebanon. The intention to stay indefinitely abroad was associated male sex and having a 2^nd ^citizenship. It was inversely associated with being a student of one of the French affiliated medical schools and a plan to train in a surgical specialty.

**Conclusion:**

An alarming percentage of students of Lebanese medical schools intend to migrate for post graduate training, mainly to the US. A minority intends to return directly to Lebanon after finishing training abroad.

## Background

The demands on human resources have been rapidly growing worldwide for a number of demographic and epidemiological conditions [1]. A major consequence has been an accelerated international migration of health workers, mainly from developing countries to more developed countries, a phenomenon known as "brain drain" [2,3]. Indeed, 23 to 28% of physicians practicing in the four major recipient countries (i.e., the United States, the United Kingdom, Canada, and Australia) are international medical graduates. The source countries for 40 to 75% of these international graduates are lower income countries and the source countries for the remainder are either middle income countries or high income countries [2].

Most of the research on the international migration of physicians has focused on low income countries, and rightly so. Indeed low income countries, affected by physician migration, unlike middle income countries, are suffering from chronic understaffing of healthcare facilities, and shrinking of available services [4]. In many low income countries, particularly in the Sub-Saharan Africa, physician migration is also a major impediment to disease-reduction initiatives sponsored by international organizations [5]. However, middle income countries affected by physician migration still lose intellectual capital and educational investment. Moreover, the dynamics of physicians' migration in middle income countries apparently differ from those in low income countries as migration has been shown to be positively related to better health systems and development in source countries. [6].

Lebanon provides an illustrative example of the flight of physicians from MICs. Lebanon has the highest emigration factor in the Middle East and North Africa and the 7th highest in the World [2]; the emigration factor is an approximate percentage of medical school graduates from a source country working in one of four major recipient countries [2]. In 2004, about 40% of those who had graduated from Lebanese medical schools over the prior 25 years were in the US [7]. Lebanon ranked 2^nd ^after adjusting for country population size among the countries from where international physicians practicing in the US graduated [7] indicating a high migration density [8].

We were interested in further exploring the dynamics of physician migration from Lebanon, as a middle income country. As residency training abroad is a critical step in the migration of physicians [9], the objective of this study was to survey students of Lebanese medical schools about their intentions to train abroad and their post training plans.

## Methods

### Participants and setting

Our target population consisted of students of Lebanese medical schools in the pre-final and final years of study. At the time of the survey, there were 6 accredited medical schools in Lebanon (see Additional file 1: Characteristics of Lebanese medical school). We excluded one of these schools as it had been recently established and did not have a pre-final or a final year class at the time. We conducted the survey during the months of October and November 2005. We intended to complete the study prior to the typical period of time during which interested students travel to the US for residency training interviews. The Institutional Review Boards of all involved institutions approved the study.

### Questionnaire

We developed the questionnaire based on the results of a qualitative study assessing the intentions and motives of students of Lebanese medical schools to train abroad [10]. We conducted the qualitative study with subjects recruited from the same target population about 4 months prior to conducting this survey. We pilot tested the questionnaire with 10 physicians who had recently graduated from Lebanese medical schools.

The questionnaire included questions relating to (See Additional file 2: Survey questionnaire):

(1) Student's demographic characteristics (age, sex, marital status, socio-economic status, having a Lebanese citizenship, and having a second citizenship) and educational characteristics (medical school, year of medical education, self-reported overall class ranking, planned residency type).

(2) Student's intentions (outcome variables of interest): intention to train abroad; the destination country; and post-training intention in terms of returning or not to Lebanon.

The questionnaire included additional questions about influencing factors and barriers to abroad training (results not reported here). All questions had a closed ended format. The questionnaire was in English as all five medical schools use English as a language of instruction to varying degrees.

### Data collection

An investigator from each of the five participating medical schools contacted eligible students at the end of a class to participate in the study. Eligible students who expressed willingness to participate filled out a paper-based survey questionnaire. The survey was anonymous and confidential. The investigator contacted eligible students on a second occasion to invite students who had not participated to consider doing so.

### Data Analysis

We first conducted a descriptive analysis of students' demographic and educational characteristics and of the outcome variables of interest (abroad training intention, destination country and post-training intention) using mean and standard deviation for continuous variables and frequencies and percentages for categorical variables.

Second, we conducted bivariable analyses using Student's t-test and the Chi-Square test to determine which demographic and educational characteristics are associated with each of the outcome variables of interest. We also evaluated whether the "post-training intention" variable was associated with the "destination country" variable.

Third, we conducted multivariable analyses to determine the factors independently associated with each of the outcome variables of interest. We collected information on all variables that we – a priori – believed might be associated with the outcome variables (i.e. the demographic and educational characteristics) and added them to the models as potential confounders. We also hypothesized that "destination county" could be independently associated with students' post-training intentions and thus used it as an independent variable for "post-training intention" variable. For the dependent variable "intention to train abroad" we used a logistic model with backward elimination and "yes, no" as the dependent variable categories. For the dependent variable "destination country" we used a multinomial model with "USA" as the references category because it was the destination country for the majority of respondents. For the dependent variable "post-training intention" we used a multinomial model with "return directly to Lebanon" as the reference category. We recoded the "post-training intention" variable into 3 categories for ease of interpretation: return directly to Lebanon, stay temporarily abroad, and stay indefinitely abroad. Finally, for each outcome variable, we assessed the impact on the results of the main regression analysis of a hierarchical regression forcing the variable "medical school" in the regression model to assess for a possible clustering within medical schools.

We considered two-sided p values and p < 0.05 as statistically significant. We used Microsoft Office Excel 2003 for data management and SPSS, version 13.0 (SPSS, Inc., Chicago, Illinois), for data analyses.

## Results

Of 576 eligible students, 430 responded to the survey. We excluded 5 responses because they were missing data for the outcome variables of interest. The survey response rate was thus 73.8%.

### Descriptive analyses

Table 1 shows the demographic and educational characteristics of respondents. Of note are the low number of respondents who were married (6(1.4%)), and who classified themselves as of lower (7(1.6%)) or upper (17(4.0%)) socio-economic status. Seventy two (16.9%) respondents had a second citizenship. Only 6 (1.4%) intended to go directly to practice without completing residency training.

**Table 1:** Baseline characteristics of respondents to a survey of pre-final and final-year students in five Lebanese Medical Schools in 2005 (N = 425; response rate 74%)

Age (years) *		Mean = 23.8; SD = 1.4
		n (%)
Sex †	Female	174 (40.9)
Marital Status ‡	Single	410 (96.5)
	Married	6 (1.4)
	Divorced	3 (0.7)
	Widow	0 (0)
Socio-economic status §	Lower	7 (1.6)
	Lower middle	101 (23.8)
	Upper middle	238 (56.0)
	Upper	17 (4.0)
Lebanese citizenship ¶	Yes	401 (94.4)
Additional citizenship **	Yes	72 (16.9%)
	Canadian	16 (22.2%)
	French	10 (13.9%)
	US	18 (25%)
	Other	28 (38.9%)
Medical school	American University of Beirut	134 (31.5)
	Beirut Arab University	51 (12.0)
	Lebanese University	113 (26.6)
	Université Saint Joseph	61 (14.4)
	University of Balamand	66 (15.5)
Year of medical school ††	Pre-final year	215 (50.6)
	Final year	207 (48.7)
Reported overall ranking in school ‡‡	Top 1/3	179 (42.1)
	Middle 1/3	193 (46.2)
	Bottom 1/3	46 (11.0)
Planned residency type§§	None (general practice)	6 (1.4)
	Surgical	137 (32.2)
	Medical	241 (56.7)
	Other	36 (8.5)

* missing n = 2;

† missing n = 3;

‡ missing n = 6;

§ missing n = 62;

¶ missing n = 3;

** missing n = 15;

†† missing n = 3;

‡‡ missing n = 7;

§§ missing n = 5;

Four hundred and six (96%) respondents intended to train abroad either for a specialty (78%) or a subspecialty (18%) (Table 2). The top 4 destination countries were the US (301(74.1%)), France (49 (12.1%)), the United Kingdom (31 (7.6%)) and Canada (17 (4.2%)) (Table 2). While 102 (25.1%) of those who intended to train abroad intended to return to Lebanon directly after finishing training, 259 (63.8%) intended to return after working abroad for a varying number of years: 142 (35.0%) for less than 5 years, 90 (22.2%) for 5 to 10 years and 27 (6.7%) for more than 10 years (Table 2). Forty three (10.6%) intended to never return to Lebanon.

**Table 2:** The plans to train abroad of pre-final and final-year students in five Lebanese Medical Schools in 2005 (N = 425; response rate 74%)

		n (%)
Plan to train abroad (N = 425)	No	19 (4.5)
	Yes, for specialty training	330 (77.6)
	Yes, for subspecialty training	76 (17.9)
Preferred training country (N = 406)	Canada	17 (4.2)
	France	49 (12.1)
	UK	31 (7.6)
	US	301 (74.1)
Post abroad training plan (N = 406) *	Return directly to Lebanon	102 (25.1)
	Work abroad < 5 years then return	142 (35.0)
	Work abroad 5–10 years then return	90 (22.2)
	Work abroad > 10 years then return	27 (6.7)
	Never return	43 (10.6)

* missing n = 2;

### Bivariable analyses

Forty percent of those who intended to train abroad were females. Fewer females than males intended to train abroad (93% vs. 97%; p = 0.047). Fewer married students intended to train abroad compared with single and divorced students (66.7% vs. 96.1% vs. 100%; p = 0.002). More students interested in medical or surgical specialties than students interested in "other specialties" (e.g. psychiatry, radiology) intended to train abroad (95.9% vs. 97.8% vs.86.1%; p = 0.02).

The destination country differed among students who had an additional citizenship by that additional citizenship: the UK (66%) for those with a British citizenship, the US (60% and 100% respectively) for those with a Canadian or a US citizenship, and France for those with a French citizenship (50%).

The post-training intentions were associated with the destination country (p < 0.001) (Table 3). Those who preferred to train in France were the most likely to intend to return to Lebanon directly (53.1%) whereas those who preferred to train in Canada were the most likely to intend to never return to Lebanon (29.4%).

**Table 3:** Post-training plans (number of years abroad) and the chosen country of abroad training of pre-final and final-year students in five Lebanese Medical Schools in 2005 (N = 425; response rate 74%)

Training Country	Return directly after training	Abroad < 5 years	Abroad 5–10 years	Abroad > 10 years	Abroad Indefinitely	Total
Canada	6 (35.3%)	2 (11.8%)	4 (23.5%)	0 (0%)	5 (29.4%)	17 (100%)
France	26 (53.1%)	14 (28.6%)	2 (4.1%)	1 (2.0%)	6 (12.2%)	49 (100%)
UK	11 (35.5%)	9 (29.0%)	3 (9.7%)	4 (12.9%)	4 (12.9%)	31 (100%)
US	58 (9.4%)	111 (37.1%)	81 (27.1%)	22 (7.4%)	27 (9.0%)	299 (100%)
Other	1 (12.5%)	6 (75.0%)	0 (0%)	0 (0%)	1 (12.5%)	8 (100%)

Total (% of all respondents)	102 (25.2%)	142 (35.1%)	90 (22.3%)	27 (6.7%)	43 (10.6%)	404 (100%)

% within country of training across rows (i.e. only percentages across rows add up to 100%)

There was a tendency for more females than males to intend to return to Lebanon directly after finishing training (31% vs. 21%; p = 0.158). More students with a Lebanese citizenship intended to return directly to Lebanon after completing abroad training compared with students without a Lebanese citizenship (26.3% vs. 4.8%; p = 0.005). More students without than students with a Lebanese citizenship intended to never return (33.3% vs. 9.5%; p = 0.005). Among students who had a second citizenship, those with a French citizenship intended to return to Lebanon directly after completing abroad training more often than students with other second citizenships (30% vs. 15% for other countries combined; p = 0.021); those with a US citizenship intended to never return to Lebanon more often than students with other second citizenships (35% vs. 12% for other countries combined; p = 0.021).

### Multivariable analyses

The factor independently associated with intention to train abroad was being single compared with being married (OR = 17.6; 95% CI 2.7–115.4) (Table 4). Forcing "medical school" variable into the model did not affect the results of the main regression analysis.

**Table 4:** Regression coefficients, p values and odds ratios of factors found significant in the multivariable analyses

**Dependent variable**	**Independent variable**	**coefficients**	**p-value**	**ORs**	**95% CI**
Intention to train abroad	Marital Status single*	2.869	0.003	17.6	2.7–115.4
Destination country Canada†	Having a second citizenship	1.745	0.007	5.7	1.6–20.4
	Being a student of the UOB‡	1.402	0.29	4.5	1.2–17.3
	a plan to train in a surgical specialty§	-2.089	0.004	0.12	0.03–0.52
	a plan to train in a medical specialty§	-2.651	< 0.001	0.07	0.17–0.29
Destination country is France†	Being a student of UL‡	1.697	0.006	5.5	1.6–18.1
	Being a student of USJ‡	2.873	< 0.001	17.7	5.4–58.4
Intention to stay abroad temporarily ¶	male sex	0.884	0.008	2.4	13–0.70
	being a student of USJ‡	-1.217	0.005	0.30	1.4–7.9
	destination country is the France†	-1.266	0.005	0.28	0.12–0.68
Intention to stay abroad indefinitely ¶	male sex	1.675	0.002	5.4	1.9–15.2
	having a 2^nd ^citizenship	2.019	0.002	7.5	2.1–27.8
	being a student of USJ‡	-1.944	0.025	0.14	0.03–0.78
	a plan to train in a medical specialty§	-1.923	0.042	0.15	0.02–0.93

* Reference category: marital status married

† Reference category: destination country is the United States

‡ Reference category: being a student of the AUB

§ Reference category: a plan to train in "other specialty"

¶ Reference category: intention to return directly to Lebanon

# Reference category: destination country is France

In terms of the destination country, the factors independently associated with the intention to train in Canada as compared to the US were: having a second citizenship (OR = 5.7; 95% CI 1.6–20.4), and being a student of the UOB (vs. AUB) (OR = 4.5; 95% CI 1.2–17.3) and a plan to train in a surgical specialty (vs. other specialty) (OR = 0.12; 95% CI 0.03–0.52) and in a medical specialty (vs. other specialty) (OR = 0.07; 95% CI 0.17–0.29) (Table 4). The factors independently associated with the intention to train in France as compared to the US were: being a student of LU (vs. AUB) (OR = 5.5; 95% CI 1.6–18.1) and being a student of USJ (vs. AUB) (OR = 17.7; 95% CI 5.4–58.4) (Table 4). Forcing "medical school" variable into the model did not impact the results of the main regression analysis.

In terms of the post-training intention, the factors independently associated with the intention to stay temporarily abroad as compared to returning directly to Lebanon after completing training were: male sex (OR = 2.4; 95% CI 1.3–4.7), being a student of USJ (vs. AUB) (OR = 0.30; 95% CI 0.13–0.70), and having the France (vs. US) as the destination country (OR = 0.28; 95% CI 0.12–0.68) (Table 4). The factors independently associated with the intention to stay indefinitely abroad as compared to returning directly to Lebanon after completing training were: male sex (OR = 5.4; 95% CI 1.9–15.2), having a 2^nd ^citizenship (OR = 7.5; 95% CI 2.1–27.8), and being a student of USJ (vs. AUB) (OR = 0.14; 95% CI 0.03–0.78), and a plan to train in a surgical specialty (vs. other specialty) (OR = 0.15; 95% CI 0.02–0.93) (Table 4). Forcing "medical school" variable into the model did not impact the results of the main regression analysis.

## Discussion

In summary, 406 (96%) of 425 students of Lebanese medical schools in the pre-final and final years responding to our survey intended to travel abroad either for specialty training (330 (77.6%)) or subspecialty training (76 (17.9%)). The top 4 destination countries were the US (301(74.1%)), France (49 (12.1%)), the United Kingdom (31 (7.6%)) and Canada (17 (4.2%)). One hundred and two (25.1%) of those who intended to train abroad intended to return to Lebanon directly after finishing training abroad; 259 (63.8%) intended to return to Lebanon after working abroad temporarily for a varying number or years; and 43 (10.6%) intended to never return to Lebanon.

This study has a number of strengths. The study population is largely representative of students of Lebanese medical schools and the high response rate decreases the likelihood of response bias. Also, this is the first study describing the demographics of students of Lebanese medical schools, an important source country for international physicians in four key recipient countries. In addition, the validity of the study questionnaire is strengthened by its thorough development. Finally, we were not able to identify any other published study that assessed, among medical students planning to train abroad, the post training migration intentions.

In terms of limitations, although the study population is representative of students of all Lebanese medical schools, it does not include students of Lebanese medical schools graduating from foreign medical schools. It is however important, from the perspective of healthcare policy development, to also assess the intentions of this group. Assessing intentions and not actual behavior represents a limitation of this study. We were not able to identify any study assessing how good of a proxy the migration intention is for the migration behavior. However, even if we were to assume a less than strong association, the percentage of those intending to train abroad remains concerning in the context of the information about the actual migration of Lebanese physicians in the recent past [7]. Another limitation of this analysis is that for some of the analyses of subgroups the confidence intervals were wide and leave us with some uncertainty about the magnitude of effect.

Recent studies from other countries have revealed high percentages of students intending to train abroad, but not as high as in our study. In a 2004 survey of 166 final year students of Indian medical schools, 59% thought of leaving India for further training abroad. The top two destinations were the US (42%) and the UK (43%). [11]. In a recent survey of first-year house officers practicing in New Zealand, 65% of the 157 respondents intended to leave New Zealand within 3 years of graduating [12]. In another New Zealand survey of final year medical students as well as to junior doctors in their first to fourth postgraduate year, 69% of respondents stated that they plan to work overseas [13]. It is estimated that up to one half of graduates of South African medical schools emigrate [14]. One study of Ugandan nursing students found that 70% of participants would like to work outside Uganda, and that within five years they would likely be working in the U.S. (59%) or the U.K. (49%) [15].

Our findings are consistent with those of the study by Arah et al. showing that source countries with better human resources for health, more economic and developmental progress, and better health status lose proportionately more physicians than the more disadvantaged countries [6]. Indeed the World Bank classifies Lebanon as a high middle income country. The physician density in Lebanon (325 physician per 100,000) is the second highest in the Middle East and North Africa [16]. In addition, and according to the 2006 World Health Report, Lebanon total expenditure on health was the highest in terms of percentage of the total gross domestic product (10.2%) and the second highest per capita (US $730) in the WHO Eastern Mediterranean Region (EMR) [17].

The extremely high percentage of students of Lebanese medical schools that intend to migrate for post graduate training is intriguing. A qualitative study exploring the factors affecting these students' intentions to train abroad found the chief motivation to be the need to gain a competitive advantage, through abroad training, in an oversaturated Lebanese job market [10]. In fact, there is abundance of medical schools and an influx of graduates of foreign medical schools leading to the relatively high physician density as noted earlier [18]. There is evidence to suggest that an oversaturation of the local physician job market, mainly by graduates of foreign medical schools, is contributing to the international migration of Lebanese medical graduates (unpublished data). Training capacity and quality were also major factors reported by respondents, 98.6% of whom intended to complete residency training. See Additional file 1: Characteristics of Lebanese medical school.

The findings of the multivariate analysis indicate that female sex was inversely associated with the intention to stay abroad temporarily or permanently. This is consistent with, although it does not completely explain, previous findings that only 17% of Lebanese medical graduates in the US are females [7], while 41% of students of Lebanese medical schools (at least in this study) are females. There is a need to explore the factors underlying these intentions and we believe that social and familial considerations are important factors. The associations of the attended medical school with the outcome variables indicate that, being a student of LU or USJ (both schools using French as a, or the, primary language of education) was associated with France being the destination countries. This illustrates the importance of the language of instruction on the destination country. Also, earlier language or cultural preference might affect both the choice of medical school and the destination country. The inverse association between the plan to train in a surgical specialty and the intention to travel abroad might be related to a perceived difficulty of obtaining surgical training spots abroad."

While some might argue that the migration of physicians from Lebanon is not a case of brain drain given the relatively high physician density (325 physician per 100,000 [19]), we believe that it is an alarming case of brain drain. First, this migration is associated with loss of intellectual capital and of educational investment with uncertain return. Second, there is indirect evidence that those who migrate might be the best among their peers [20,21]. This possible selection process could negatively affect the quality of health care services in Lebanon. Unfortunately, we were not able to identify studies of the impact of physician migration on the Lebanese healthcare system.

From the perspective of the US physician workforce, the impact is probably less dramatic given the relative size of the US workforce (902,053 physicians in 2006) [22]. However, US policymakers should consider the implications of the global migration of health workers for the source countries, LICs in particular. Although these countries benefit financially through remittances, skills transfer, and possible investment upon migrants' return [23], they suffer from a loss of educational investment and intellectual capital, [4] and from worsening of the already depleted healthcare resources in these countries [24]. A major consequence is the widening of the gap in health inequities worldwide [24].

## Conclusion

The findings of this study are alarming in terms of the future migration of Lebanese physicians and have implications for both policy making and future research. Lebanese policy makers need to devise a comprehensive national health workforce plan using the proposed strategies and frameworks to deal with physician migration [25,26]. Such a plan should carefully address the oversaturation of the Lebanese job market and other factors contributing to the migration of physicians, such as residency training capacity and quality.

There is a need for research investigating the impact of migration on the effectiveness and efficiency of health care systems in both the source and receiving countries. There is also a need for further methodological work in the field of human resources for health [8]. We plan to follow up this cohort of students to compare their current intentions with their future actual behavior in terms of country of residency training and most importantly in terms of migration status.

## Abbreviations

None

## Competing interests

The authors declare that they have no competing interests.

## Authors' contributions

**EAA: **conception and design, data collection, data analysis, data interpretation, article drafting, final approval of the article.** NM: **conception and design, data interpretation, critical revision and final approval of the article.** SM: **data collection, critical revision and final approval of the article.** CA: **data collection, critical revision and final approval of the article. **AA: **data collection, critical revision and final approval of the article.** JC: **data collection, critical revision and final approval of the article.** MS: **data collection, critical revision and final approval of the article. **CKL: **data interpretation, critical revision and final approval of the article.** BJBG: **data interpretation, data analysis, critical revision and final approval of the article. **HJS: **conception and design, data analysis, data interpretation, critical revision and final approval of the article.

## Pre-publication history

The pre-publication history for this paper can be accessed here:



## Supplementary Material

Additional file 1 Characteristics of Lebanese medical school Click here for file

Additional file 2 Survey questionnaire Click here for file
