# Supplementary material for: Post-graduation migration intentions of students of Lebanese medical schools: a survey study
Source: BMC Public Health. 2008 Jun 2;8:191. doi: 10.1186/1471-2458-8-191 (PMC2424042; doi:10.1186/1471-2458-8-191)
Supplement: Additional file 1 — Characteristics of Lebanese medical school [file 1471-2458-8-191-S1.doc]

**Additional file 1:** Characteristics of Lebanese medical schools

|  | **Year instruction began** | **Admission requirement** | **Official language(s) of instruction** | **Length of studies (years)** | **Degree granted** | **Type** |
| --- | --- | --- | --- | --- | --- | --- |
| **American University of Beirut (AUB)** | 1868 | Bachelor degree | English | 4 | Doctor of Medicine (M.D.) | Private |
| **Université Saint Joseph (USJ)** | 1883 | High school graduation | French | 7 | Doctor of Medicine (M.D.) | Private |
| **Lebanese University (LU)** | 1983 | High school graduation | English, French | 7 | Doctor of Medicine (M.D.) | Public |
| **Beirut Arab University (BAU)** | 1995 | High school graduation | English | 6 | Bachelor degree in Medicine and Surgery (M.B.B.Ch.) | Private |
| **University of Balamand (UOB)** | 2000 | Bachelor degree | English | 4 | Doctor of Medicine (M.D.) | Private |
| **Holy Spirit University of Kaslik (USEK)** | 2002 | High school graduation | English, French | 7 | Doctor of Medicine (M.D.) | Private |
| [**Lebanese American University**](http://www.lau.edu.lb/) **(LAU)** | 2009 | Bachelor degree | English | 4 | Doctor of Medicine (M.D.) | Private |

Note: there is no mandatory post graduation period of health service employment in Lebanon
