# Supplementary material for: Post-graduation migration intentions of students of Lebanese medical schools: a survey study
Source: BMC Public Health. 2008 Jun 2;8:191. doi: 10.1186/1471-2458-8-191 (PMC2424042; doi:10.1186/1471-2458-8-191)
Supplement: Additional file 2 — Survey questionnaire [file 1471-2458-8-191-S2.doc]

Additional file 2: Survey questionnaire

***Student Characteristics***

1. **Your age** (Please circle the right answer)

<21 21 22 23 24 25 26 27 28 29 30

31 32 33 34 35 36 37 38 39 40 >40

1. **Gender**

🞏 1. Female 🞏 2. Male

1. **Marital status**

🞏 1. Single 🞏 2. Married 🞏 3. Divorced 🞏 4. Widow

1. **Religion: _____________________**
2. **Lebanese Citizenship**

🞏 1. Yes 🞏 2. No. Citizenship: ______________

1. **Do you have a 2^nd^ citizenship or permanent residency?**

🞏 1. No 🞏 2. Yes. Country: ­­­­­­­­­­­­­­­­­­­________________

1. **Year of Medical School**

🞏 1. Pre-final year 🞏 2. Final year

1. **Your estimated overall ranking in class**

🞏 1. Top 1/3 🞏 2. Middle 1/3 🞏 3. Bottom 1/3

1. **What type of residency training do you plan to do?** *(check only one)*

🞏 1. None. I desire to be a general practitioner 🞏 2. Surgical

🞏 3. Medical 🞏 4. Other: __________

1. **How would you rate your socio-economic status?**

🞏 1. Lower 🞏 2. Lower middle 🞏 3. Upper middle 🞏 4. Upper

***Abroad training***

1. **Do you intend to do your training abroad?**

🞏 1. No

🞏 2. Yes, for specialty training

🞏 3. Yes, for subspecialty training, after completing specialty training in Lebanon

1. **If “yes” to q.10, what country would be your first choice?** *(check only one)*

🞏 1. United Kingdom 🞏 2. Canada

🞏 3. France 🞏 4. United States 🞏 5. Other: __________

1. **If “yes” to q.10, what is your intention after you finish training abroad?** *(check only one)*

🞏 1. Return directly to Lebanon

🞏 2. Work abroad for less than 5 years then return to Lebanon

🞏 3. Work abroad for 5-10 years then return to Lebanon

🞏 4. Work abroad for more than 10 years then return to Lebanon

🞏 5. Never return to Lebanon

1. **If “yes” to q.10, which is the most important goal for you to achieve through training abroad** *(check only one)*

🞏 1. Excel professionally

🞏 2. Prosper financially

🞏 3. Establish myself quickly

🞏 4. Achieve long-term stability and security

🞏 5. Keep options open in terms of working in or outside Lebanon

🞏 6. Obtain the citizenship of the country of training

🞏 7. Other:___________________­­­­­­­­­­­­­­­­­­­­­­­­­­­­­­­­­________

1. **What are the barriers for you to train abroad?** *(check all that apply)*

🞏 1. Required process of certification (exams and tests)

🞏 2. Expenses (exams, applications, traveling)

🞏 3. Getting visas

🞏 4. Other:___________________­­­­­­­­­­­­­­­­­­­­­­­­­­­­­­­­­­­­­­­­­­­­­­­­­­­________

***Influencing Factors***

1. **Do you have family members or friends living abroad who would assist you if you travel to train abroad?**

🞏 1. Yes 🞏 2. No

1. **Are there doctors in your university that encourage you to train abroad?**

🞏 1. Yes 🞏 2. No

1. **Are there doctors in your university that would assist you to travel and train abroad?**

🞏 1. Yes 🞏 2. No

1. **Does witnessing residents traveling to retrain abroad motivate you to train abroad yourself?**

🞏 1. Yes 🞏 2. No

1. **Have you completed an observership or an elective rotation abroad?**

🞏 1. Yes, country: ­­­­­­­­­­­­­­­­­­­­­­­­­­­­­ ______________

🞏 2. No, but I am planning to, Country: ­­­­­­­______________

🞏 3. No, and I am not planning to

1. **From where did you get your information about training abroad?** *(check all that apply)*

🞏 1. Media (movies, TV series)

🞏 2. Reports by family members or friends living abroad

🞏 3. Reports by medical students who completed observership or elective abroad

🞏 4. Reports by doctors who trained or are training abroad

🞏 5. By comparing doctors trained locally to those trained abroad

🞏 6. My own observations while on observership or elective abroad

🞏 7. Other:___________________­­­­­­­­­­­­­­­­­­­­­­­­­­­­­­­­­________

1. **Do you agree that the Lebanese society expects you to train abroad? *(circle one number)***

| - 3 | - 2 | -1 | 0 | +1 | +2 | +3 |
| --- | --- | --- | --- | --- | --- | --- |
| I strongly disagree |  |  | I neither agree or disagree |  |  | I strongly agree |

1. **Which of the following groups in the population consider doctors trained abroad as more qualified than doctors trained in Lebanon?** *(check all that apply)*

🞏 1. General public 🞏 2. Patients

🞏 3. Academic community 🞏 4. Healthcare institutions

🞏 5. I personally consider so 🞏 6. None of the above

1. Rate the impact of each of the factors listed below on your motivation to train abroad using the following scale:

| - 3 | - 2 | -1 | 0 | +1 | +2 | +3 |
| --- | --- | --- | --- | --- | --- | --- |
| I strongly disagree |  |  | I neither agree or disagree |  |  | I strongly agree |

*Factors related to residency training*

| 1. **Residency training opportunities**   Availability of desired specialty, intensity & fairness of competition | -3 -2 -1 0 +1 +2 +3 |
| --- | --- |
| 1. **Clinical training**   Exposure to cases, learning procedures, autonomy, application of theoretical learning | -3 -2 -1 0 +1 +2 +3 |
| 1. **Research training**   Financial resources, mentorship, possibility to publish | -3 -2 -1 0 +1 +2 +3 |
| 1. **Teaching in residency programs**   Doctors’ and programs’ commitment to teaching, presence of a curriculum | -3 -2 -1 0 +1 +2 +3 |
| 1. **Working conditions of residents**   Amount of work, relationship with doctors and nurses, rewarding and evaluation systems | -3 -2 -1 0 +1 +2 +3 |
| 1. **Financial situation of residents**   Income, financial independency, ability to start a family | -3 -2 -1 0 +1 +2 +3 |
| 1. **Impact of residency training on future career**   Ability to enter job market in Lebanon or abroad, chance of an academic career | -3 -2 -1 0 +1 +2 +3 |

*Factors related to working as a doctor*

| 1. **Job opportunities**   Intensity and fairness of competition, academic career opportunity, ability to work in your specialty of training | -3 -2 -1 0 +1 +2 +3 |
| --- | --- |
| 1. **Working conditions of doctors**   Amount of work, professional standards, advancement, continuous medical education | -3 -2 -1 0 +1 +2 +3 |
| 1. **Financial situation of doctors**   Income, stability of income | -3 -2 -1 0 +1 +2 +3 |

*Other factors*

| 1. **Personal conditions**   Issues related to partner, parents, children | -3 -2 -1 0 +1 +2 +3 |
| --- | --- |
| 1. **Social conditions**   Social norms, social system, social relationships, social and family support, lifestyle, living dependently or independently | -3 -2 -1 0 +1 +2 +3 |
| 1. **Political conditions**   Political situation, political system, ability to make changes, personal security | -3 -2 -1 0 +1 +2 +3 |

1. Any comments? ___________________________________________________________
